# National differences in implementation of minimally invasive surgery for colorectal cancer and the influence on short-term outcomes

**DOI:** 10.1007/s00464-021-08974-1

**Published:** 2022-03-08

**Authors:** A. K. Warps, D. Saraste, M. Westerterp, R. Detering, A. Sjövall, A. Martling, J. W. T. Dekker, R. A. E. M. Tollenaar, P. Matthiessen, P. J. Tanis, The Dutch ColoRectal Audit and Swedish Colorectal Cancer Registry

**Affiliations:** 1 Department of Surgery, Leiden University Medical Centre, Albinusdreef 2, 2333 ZA Leiden, Netherlands; 2 Dutch Institute for Clinical Auditing, Rijnsburgerweg 10, 2333 AA Leiden, Netherlands; 3 Department of Surgery, Södersjukhuset, 118 83 Stockholm, Sweden; 4 Department of Molecular Medicine and Surgery, Karolinska Insitutet, 171 76 Stockholm, Sweden; 5 Department of Surgery, Haagland Medisch Centrum, Lijnbaan 32, 2512 VA Den Haag, Netherlands; 6 Department of Surgery, Amsterdam University Medical Centres, Meibergdreef 9, 1105 AZ Amsterdam, Netherlands; 7 Department of Surgery, Karolinska University Hospital, Anna Steckséns gata 53, 171 64 Solna, Sweden; 8 Department of Surgery, Reinier de Graaf Groep, Reinier de Graafweg 5, 2625 AD Delft, Netherlands; 9 Department of Surgery, Örebro University Hospital, von Rosens väg 1, 70185 Örebro, Sweden; 10 Department of Surgery, Faculty of Medicine and Health Sciences, Örebro University, 70182 Örebro, Sweden; 11 Department of Surgery, Cancer Centre Amsterdam, Amsterdam University Medical Centres, University of Amsterdam, De Boelelaan 1117, 1081 HV Amsterdam, Netherlands

**Keywords:** Colorectal cancer, Minimal invasive surgery, Laparoscopy, Robotic surgery, Hospital volume, Short-term outcomes

## Abstract

**Background:**

The timing and degree of implementation of minimally invasive surgery (MIS) for colorectal cancer vary among countries. Insights in national differences regarding implementation of new surgical techniques and the effect on postoperative outcomes are important for quality assurance, can show potential areas for country-specific improvement, and might be illustrative and supportive for similar implementation programs in other countries. Therefore, this study aimed to evaluate differences in patient selection, applied techniques, and results of minimal invasive surgery for colorectal cancer between the Netherlands and Sweden.

**Methods:**

Patients who underwent elective minimally invasive surgery for T1-3 colon or rectal cancer (2012–2018) registered in the Dutch ColoRectal Audit or Swedish ColoRectal Cancer Registry were included. Time trends in the application of MIS were determined. Outcomes were compared for time periods with a similar level of MIS implementation (Netherlands 2012–2013 versus Sweden 2017–2018). Multilevel analyses were performed to identify factors associated with adverse short-term outcomes.

**Results:**

A total of 46,095 Dutch and 8,819 Swedish patients undergoing MIS for colorectal cancer were included. In Sweden, MIS implementation was approximately 5 years later than in the Netherlands, with more robotic surgery and lower volumes per hospital. Although conversion rates were higher in Sweden, oncological and surgical outcomes were comparable. MIS in the Netherlands for the years 2012–2013 resulted in a higher reoperation rate for colon cancer and a higher readmission rate but lower non-surgical complication rates for rectal cancer if compared with MIS in Sweden during 2017–2018.

**Conclusion:**

This study showed that the implementation of MIS for colorectal cancer occurred later in Sweden than the Netherlands, with comparable outcomes despite lower volumes. Our study demonstrates that new surgical techniques can be implemented at a national level in a controlled and safe way, with thorough quality assurance.

**Supplementary Information:**

The online version contains supplementary material available at 10.1007/s00464-021-08974-1.

In 2018, over 1.8 million new cases of colorectal cancer and 881,000 deaths due to colorectal cancer were estimated worldwide [1]. Therefore, it is essential that patients receive the best quality of care to optimize short- and long-term outcomes. For this reason, nationwide cancer registries have been initiated, which monitor the current healthcare process and provide benchmarked information to healthcare providers to improve the quality of care [2, 3]. The Swedish ColoRectal Cancer Registry (SCRCR), initiated in 1995, and the Dutch ColoRectal Audit (DCRA), set up in 2009 [4], are two clinical audits that started as pure surgical quality registries. Nowadays, both audits strive to monitor quality of the complete multidisciplinary colorectal cancer care [5].

At an international level, significant differences in colorectal cancer care exist between countries. Therefore, international comparisons of treatment strategies and results with data from national audits can provide valuable insights into national performance and, as a consequence, potential areas for country-specific evaluation and improvement [6–8].

During the past decades, colorectal cancer surgery has substantially changed [4], with the introduction of minimally invasive surgery (MIS) as an important innovation. MIS has revealed better postoperative recovery and even a reduction in mortality at a population level [9–17]. The degree and speed of implementation of MIS vary substantially among European countries [18]. In the Netherlands, laparoscopic surgery has become the standard surgical approach in colorectal cancer surgery, with full implementation in every hospital. In Sweden, adoption of MIS for colorectal cancer occurred relatively late, and implementation has still not reached a level of 90% implementation. In contrast to the Netherlands, a large proportion of the minimally invasive rectal cancer resections is performed by a robot-assisted laparoscopic approach in Sweden [19–22].

Analysing differences in implementation of new surgical techniques at a national level can provide valuable information for surgeons and policy makers as part of the plan-do-check-act cycle, and might help in moving forward with similar implementation programs in other countries. Therefore, this international collaborative study aimed to evaluate the differences in patient selection, applied techniques and short-term outcomes of MIS for colon- and rectal cancer between two Northern European countries with a different degree and speed of implementation of MIS.

## Methods

This population-based observational cohort study was performed with pseudonymized data from two nationwide colorectal cancer registries (SCRCR and DCRA). Both audits have a nationwide coverage with reported data completeness in the DCRA of > 95% and in the SCRCR of > 98%, besides high validity of the data [2, 23]. According to national law for the Netherlands, no ethical approval or informed consent was required, whereas ethical permission was obtained from the Swedish Authority for Ethical Approval (registration number 2015/906–31/1 and 2020–01335). To determine the degree and speed of MIS implementation, all patients who were registered in the DCRA or SCRCR after elective resection for a first solitaire primary colon- or rectal cancer by either open approach or any MIS technique between January 1st 2012 and December 31st, 2018, were included. Subsequently, all patients with a clinical T4-stage and those who underwent emergency resection were excluded since locally advanced and emergency cases are still considered relative contraindications for MIS and would introduce more heterogeneity within the study population. Multivisceral resection was not an exclusion criterion if performed for cT1-3 colorectal cancer, and neither was unknown clinical T-stage (cTx). In addition, patients with stage IV disease that underwent resection of their primary tumour were included.

### Data extraction, outcomes, and definitions

The following variables were extracted from the DCRA and SCRCR database: patient- and disease characteristics, procedural characteristics and postoperative outcomes within 30 days after surgery or during primary admission. In the SCRCR, the short-term postoperative follow-up duration is 30-days, although the total follow-up time is 5-years. In the DCRA, the 30-day follow-up was registered until 2017, and this was extended to 90 days since January 1st, 2018. Since long-term information is not collected for the DCRA, the 30-day outcomes were reported for both countries. The SCRCR registered the applied technique of MIS during the whole study period. The DCRA recorded robot-assisted surgery only since January 1st, 2018, but had already been introduced in a few centres in the preceding years. Annual hospital volume was extracted for each centre based on the number of elective MIS procedures for T1-3 colorectal cancer performed per hospital per year.

The primary outcome measures were completeness of resection (all resection margins > 1 mm), complications (categorized in non-surgical complications, surgical complications, and both non-surgical and surgical complications), reoperation, readmission, and postoperative mortality. Secondary endpoints included conversion rate, proportions of lymph node count ≥ 12, and positive lymph nodes. Non-surgical complications included pulmonary-, cardiac-, thromboembolic-, infectious-, neurological complications, and unspecified non-surgical complications. Surgical complications consisted of anastomotic leakage, fascial dehiscence, haemorrhage, intra-abdominal infection (e.g., abscess, bowel perforation, ureter/bladder perforation), wound infection, and unspecified surgical complications (e.g., ileus, stoma complications).

The type of surgical procedure for colon cancer was categorized into three groups: a right-sided resection group, including ileocecal resections, (extended) right hemicolectomies and transversectomies, a left-sided resection group, including (extended) left hemicolectomies, sigmoid/anterior resections, Hartmann procedures, and a (sub)total colectomy group. Rectal cancer procedures were categorized as anterior resection with primary anastomosis with or without diverting stoma, Hartmann's procedure, and abdominoperineal excision (APE). For the evaluation of hospital volume, hospitals were categorized into low, low-intermediate, intermediate-high, and high volume hospitals, which was defined as < 30, 30–60, 61–90, and > 90 for colon cancer resections and < 12, 12–25, 26–50, > 50 for rectal cancer resection (as defined by Detering et al. [8]).

### Data analysis

Patients were stratified for country and colon or rectal cancer. Categorical or dichotomous variables were represented as absolute numbers of cases and percentages. Time-trends in hospital volume, type of MIS (including conventional laparoscopy and robot-assisted laparoscopy), and conversion rates were analysed for each year and displayed in figures. In the overall analysis, all eligible patients from both countries were included for the whole study period. In a subgroup analysis, outcomes of MIS were compared during two separate 2-year periods where the two countries had a similar level of implementation of MIS, based on the analysis of the proportion of MIS over time (Netherlands 2012–2013 and Sweden 2017–2018). A Pearson Chi-square test was used to assess significance.

Multilevel logistic regression analyses were used to assess factors associated with incomplete resection margin, overall complications, reoperation, and readmission. A multilevel regression analysis was used to provide a more accurate estimate than ordinary logistic regression analyses when dealing with potential hierarchically structured, i.e., clustered data, since dependency of patients in hospitals is taken into account [24, 25]. Incomplete resection was adjusted for neoadjuvant therapy, year of surgery, approach, procedure type, multivisceral resection, TNM-stage, and hospital volume. All other outcomes were adjusted for sex, BMI, age, ASA-score, multivisceral resection, T-stage, M-stage and neoadjuvant therapy, procedure type, surgical approach, year of surgery, and hospital volume. For neoadjuvant treatment, chemotherapy was added to the model for colon cancer, and radiotherapy was added for rectal cancer. The pathological T-stage and N-stage were included for colon cancer in the multilevel analyses, whereas the clinical stage was included for rectal cancer due to differences in the reliability of clinical tumour and lymph node staging on radiologic imaging (e.g., CT-imaging for colon cancer and MRI for rectal cancer) and the use of neoadjuvant treatment for down-staging (rarely in colon cancer and frequently in rectal cancer).

Multicollinearity was assessed with the variance of inflation factor (VIF). In case of a VIF of > 2.5 was found, it was considered as multicollinear, and as a consequence, one of the variables was excluded. Results are reported as adjusted odds ratio (AOR) with 95% confidence intervals (95% CI). A p-value of < 0.05 was considered significant. All analyses were performed in Rstudio version 1.4.1106 (2021).

## Results

### MIS implementation

**Fig. 1:**
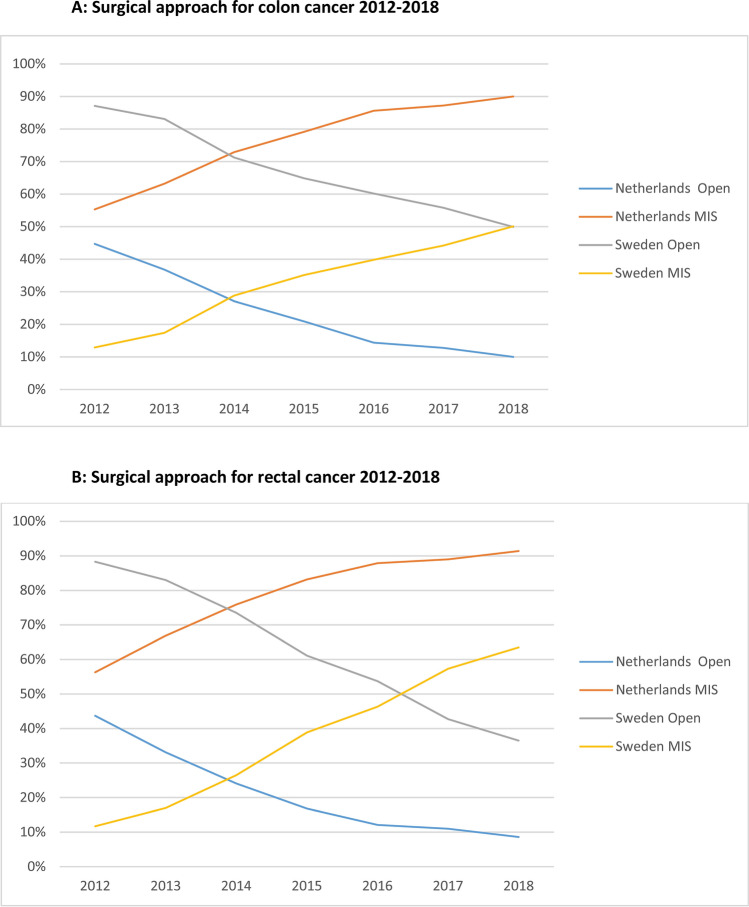
**A** Time trend (2012–2018) of elective open vs. minimally invasive surgery for colon cancer in the Netherlands and Sweden. **B** Time trend (2012–2018) of elective open vs. minimally invasive surgery for rectal cancer in the Netherlands and Sweden

In 2012–2018, 42,581 and 19,146 patients underwent an elective resection for primary colon- and rectal cancer in the Netherlands using either an open or laparoscopic approach. Corresponding numbers were 18,407 and 9090 patients in Sweden. The annual proportion of MIS for colon- and rectal cancer in the Netherlands and Sweden during the study period is depicted in Fig. 1. A predominance in using MIS in colorectal cancer was reached approximately 5 years later in Sweden than in the Netherlands, with a similar speed of implementation based on the more or less parallel curves.

### Hospital volume, technique, and conversion

A total of 54,914 patients that underwent elective MIS for T1-3 colon- or rectal cancer between 2012 and 2018 were included for final analyses. The Dutch study population consisted of 46,095 patients, of which 31,853 underwent MIS for colon cancer and 14,242 for rectal cancer. Of the total 8819 patients who underwent MIS in Sweden, 5834 had colon cancer, and 2985 had rectal cancer.

**Fig. 2:**
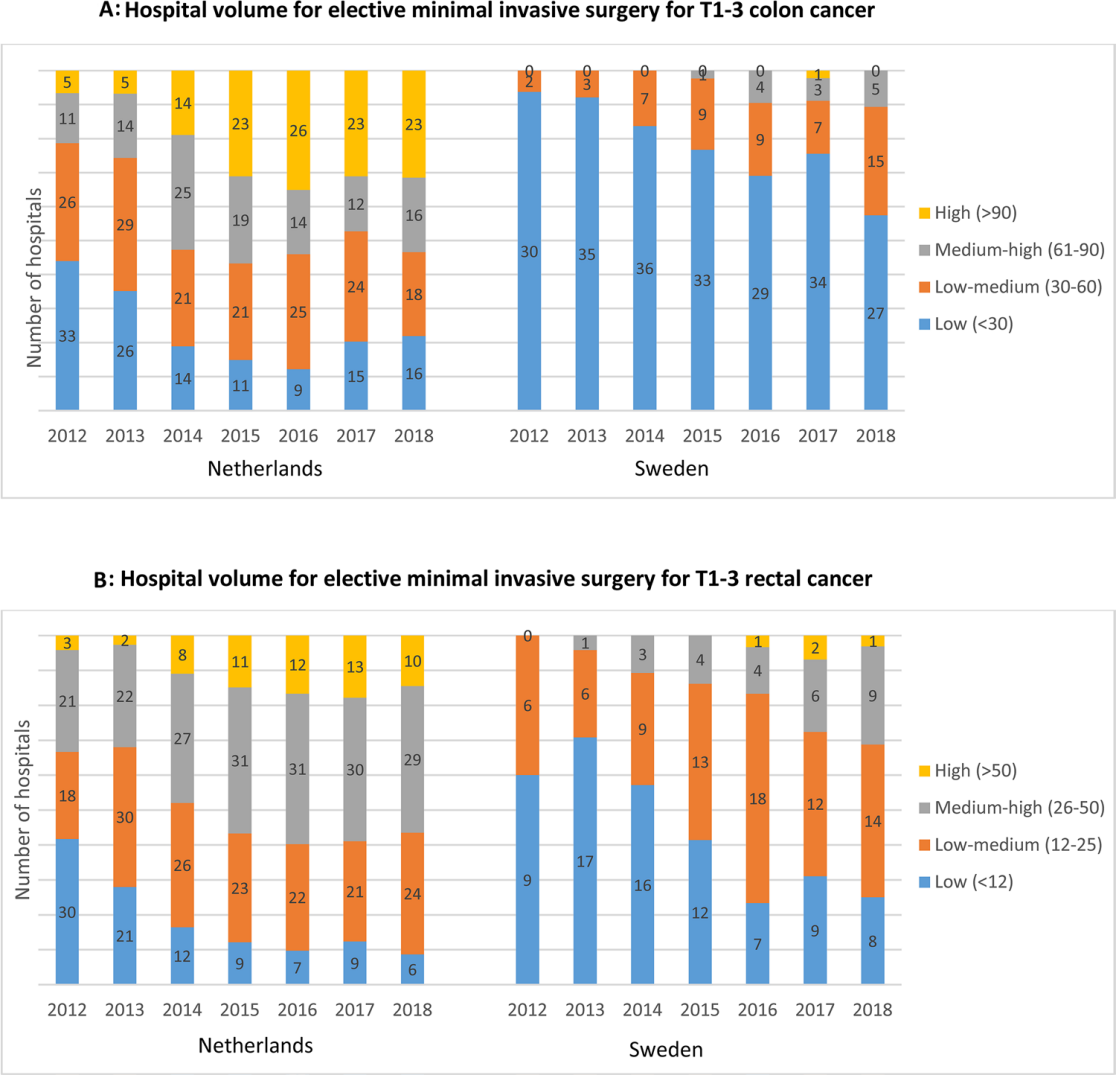
**A** Number of hospitals performing elective minimal invasive surgery in Sweden and the Netherlands, categorized in four hospital volume groups, during 2012–2018. **B** Number of hospitals performing elective minimal invasive surgery in Sweden and the Netherlands, categorized in hospital volume groups, during 2012–2018

Figure 2 shows the hospital volume of elective MIS procedures for T1-3 colon (Fig. 2A) and rectal (Fig. 2B) cancer. The number of hospitals performing elective MIS for T1-3 colon and rectal cancer increased in Sweden, whereas the number of hospitals for rectal cancer decreased in the Netherlands. Besides, more intermediate-high and high volume hospitals were performing MIS for colon and rectal cancer in the Netherlands compared to Sweden. A trend towards less low volume MIS hospitals for colon cancer was observed in Sweden over the years (*N* = 30 in 2012 and *N* = 27 in 2018). In the Netherlands, most MIS procedures were performed in high-volume centres for colon cancer (44.4%) and intermediate-high volume hospitals for rectal cancer (48.2%), while in Sweden, most MIS colon cancer resections were performed in low volume hospitals (44.3%) and low-intermediate hospitals for rectal cancer (47.5%) (Table 1).

**Table 1:** Baseline characteristics of Dutch and Swedish patients who underwent elective MIS for T1-3 colorectal cancer during 2012–2018

	Colon cancer (*N* = 37,687)		Rectal cancer (*N* = 17,227)	
	Netherlands (*N* = 31,853)	Sweden (*N* = 5834)	Netherlands (*N* = 14,242)	Sweden (*N* = 2985)
Preoperative characteristics				
Age (years)				
< 60	4622 (14.5)	768 (13.2)	3120 (21.9)	566 (19.0)
60–70	10,102 (31.7)	1436 (24.6)	4900 (34.4)	906 (30.4)
70–80	11,696 (36.7)	2075 (35.6)	4534 (31.8)	1097 (36.8)
≥ 80	5428 (17.0)	1555 (16.7)	1658 (11.8)	416 (13.9)
Missing	5	0	3	0
Sex				
Male	17,291 (54.3)	2839 (48.7)	9145 (64.2)	1730 (58.0)
Female	14,554 (45.7)	2995 (51.3)	5094 (35.8)	1255 (42.0)
Missing	8	0	3	0
BMI (kg/m^2^)				
< 30	24,933 (79.5)	5409 (92.5)	11,539 (82.6)	2794 (93.6)
≥ 30	6429 (20.5)	425 (7.5)	2432 (16.3)	191 (6.4)
Missing	20	0	7	0
ASA score				
I-II	24,380 (76.5)	3967 (68.0)	11,819 (83.0)	2305 (77.2)
III +	7468 (23.4)	1780 (30.5)	2421 (17.0)	638 (21.4)
Missing	5	87	2	42
Tumour characteristics				
cT stage				
cT1-2	5418 (17.0)	1935 (33.2)	4507 (31.6)	1091 (36.5)
cT3	6922 (21.7)	2563 (43.9)	9158 (64.3)	1812 (60.7)
cTx	19,513 (61.3)	1336 (22.9)	577 (4.1)	82 (2.8)
cN stage				
cN0	12,172 (38.2)	3527 (60.5)	6666 (46.8)	1411 (47.3)
cN1-2	4228 (13.3)	1793 (30.7)	7104 (49.9)	1512 (50.7)
cNx	15,453 (48.5)	514 (8.8)	472 (3.3)	62 (2.1)
Location				
Ascending colon	13,511 (42.4)	3067 (52.6)	–	–
Transverse colon	2892 (9.1)	200 (3.4)	–	–
Descending colon	1919 (6.0)	180 (3.1)	–	–
Sigmoid	13,531 (42.5)	–	–	–
Missing	9	–	–	–
Distance from anal verge				
≤ 5 cm	–	–	4805 (33.7)	786 (26.3)
6–10 cm	–	–	5253 (36.9)	1264 (42.3)
> 10 cm	–	–	–	917 (30.7)
Missing	–	–	–	73
Preoperative work-up				
Preoperative MDT*				
No	1302 (4.8)	223 (3.8)	70 (0.6)	12 (0.4)
Yes	25,423 (94.6)	5585 (95.7)	11,962 (98.8)	2972 (99.6)
Missing	149	26	77	1
Neoadjuvant radiotherapy				
No	–	–	5699 (40.0)	1379 (46.2)
SCRT	–	–	4457 (31.3)	1316 (44.1)
CRT	–	–	3902 (27.4)	217 (7.3)
Other RTx scheme	–	–	177 (1.2)	73 (2.4)
Missing	–	–	7	0
Neoadjuvant chemotherapy				
No	30,967 (97.2)	5787 (99.2)	–	–
Yes	249 (0.8)	47 (0.8)	–	–
Missing	637	0	–	–
Surgical characteristics				
Year of surgery				
2012–2015	16,218 (50.9)	2275 (39.0)	7385 (51.9)	1097 (46.8)
2016–2018	15,635 (49.1)	3559 (61.0)	6857 (48.1)	1888 (63.2)
Hospital volume				
Low	2385 (7.5)	2857 (44.3)	587 (4.1)	350 (11.7)
Low-intermediate	7028 (22.1)	2284 (39.1)	3080 (21.6)	1418 (47.5)
Intermediate-high	8290 (26.0)	867 (14.9)	6864 (48.2)	985 (33.0)
High	14,150 (44.4)	96 (1.6)	3711 (26.1)	232 (7.8)
Approach				
Laparoscopic	31,590 (99.2)	5175 (88.8)	13,830 (97.1)	1492 (50.0)
Robot-assisted	263 (0.8)	659 (11.3)	412 (2.9)	1493 (50.0)
MIS Converted	3327 (10.4)	1018 (17.4)	1198 (8.4)	432 (14.5)
Procedure				
Ileocecal resection	166 (0.5)	12 (0.2)	–	–
Right hemicolectomy	14,130 (44.4)	3119 (53.5)	–	–
Transversectomy	529 (1.7)	22 (0.4)	–	–
Left hemicolectomy	3495 (11.0)	350 (6.0)	–	–
(Sub)total colectomy	332 (1.0)	73 (1.3)	–	–
Sigmoid/anterior resection	12,355 (38.8)	2191 (37.6)	9177 (64.4)	1682 (56.3)
Hartmann	801 (2.5)	67 (1.1)	1852 (13.0)	221 (7.4)
Abdominoperineal excision	–	–	3165 (22.2)	1082 (36.2)
Other	45 (0.1)	0 (0.0)	48 (0.3)	0 (0.0)
Multivisceral resection				
No	30,081 (94.4)	5615 (96.2)	13,721 (96.3)	2856 (95.7)
Yes	1255 (3.9)	219 (3.8)	297 (2.1)	129 (4.3)
Missing	517	0	224	0
Stoma**				
No	30,104 (94.5)	5505 (94.4)	4767 (42.4)	409 (21.4)
Diverting-stoma	542 (1.7)	129 (2.2)	4499 (40.1)	1237 (64.9)
End-stoma	1157 (3.6)	182 (3.1)	1952 (17.4)	258 (13.5)
Missing	517	18	15	3
Tumour characteristics				
(y)pT stage				
(y)pT0-1	4446 (14.0)	654 (11.2)	2712 (19.0)	436 (14.6)
(y)pT2	6941 (21.8)	1204 (20.6)	4773 (33.5)	1015 (34.0)
(y)pT3	17,242 (54.1)	3221 (55.2)	6348 (44.6)	1409 (47.2)
(y)pT4	2926 (9.2)	709 (12.2)	287 (2.0)	88 (2.9)
(y)pTx	298 (0.9)	46 (0.8)	122 (0.9)	37 (1.2)
(y)pN stage				
(y)pN0	20,373 (64.0)	3727 (63.9)	9410 (66.1)	1092 (63.7)
(y)pN1	7466 (23.4)	1433 (24.6)	3354 (23.6)	152 (5.1)
(y)pN2	3706 (11.6)	590 (12.2)	1364 (9.6)	275 (9.2)
(y)pNx	308 (1.0)	84 (1.4)	114 (0.8)	41 (1.4)
M stage				
M-	29,947 (94.0)	5545 (95.1)	13,489 (94.7)	2833 (94.9)
M1	1906 (6.0)	287 (4.9)	753 (5.3)	152 (5.1)
Postoperative outcomes				
Number of lymph nodes				
< 12	2262 (7.1)	383 (6.6)	2016 (14.2)	334 (11.2)
≥ 12	29,497 (92.6)	5356 (91.8)	12,203 (85.7)	2601 (87.1)
Missing	94	95	23	50
Positive lymph nodes				
No	20,559 (64.5)	3862 (66.2)	9525 (66.9)	2008 (67.3)
Yes	11,011 (34.6)	1860 (31.9)	4620 (32.4)	916 (30.7)
Missing	283	112	97	61
Resection margin				
Complete	31,374 (98.5)	5599 (96.0)	13,620 (95.6)	2781 (93.2)
Incomplete	239 (0.8)	97 (1.7)	602 (4.2)	204 (6.8)
Missing	240	138	20	0
Complication				
No	24,417 (76.7)	4677 (80.2)	9475 (66.5)	1870 (62.6)
Non-surgical	2705 (8.5)	458 (7.9)	344 (11.3)	341 (11.4)
Surgical	2922 (9.2)	535 (9.2)	2033 (14.3)	616 (20.6)
Non-surgical & surgical	1809 (5.7)	146 (2.5)	1233 (8.6)	144 (4.8)
Missing	0	18 (0.3)	0	14
Reoperation				
No	29,642 (93.1)	5453 (93.5)	12,945 (90.9)	2718 (91.1)
Yes	2132 (6.7)	359 (6.2)	1262 (8.9)	256 (8.6)
Missing	79	22	35	11
Readmission				
No	29,242 (91.8)	5453 (93.5)	12,047 (84.6)	2612 (86.0)
Yes	1977 (6.2)	359 (6.2)	1899 (13.3)	409 (13.5)
Missing	634	22	296	16
Mortality***				
No	31,432 (98.7)	5750 (98.6)	14,090 (98.9)	2954 (99.0)
Yes	419 (1.3)	57 (1.0)	151 (1.1)	15 (0.5)
Missing	2	27	1	16

Missing values of less than 10% are only reported as numbers in this table

*Preoperative MDT was registered for 2012–2017 in the Netherlands (*N* = 26,87 for colon cancer and *N* = 12,109 for rectal cancer) and therefore analysed for this period

**Rectal cancer patients that underwent an APE were excluded from the analyses of stoma rate [*N* = 3009 (NL) and *N* = 1907 (SE)]

***Swedish patients labelled as missing for mortality, represent patient who are lost to follow-up due to official emigration from Sweden

**Fig. 3:**
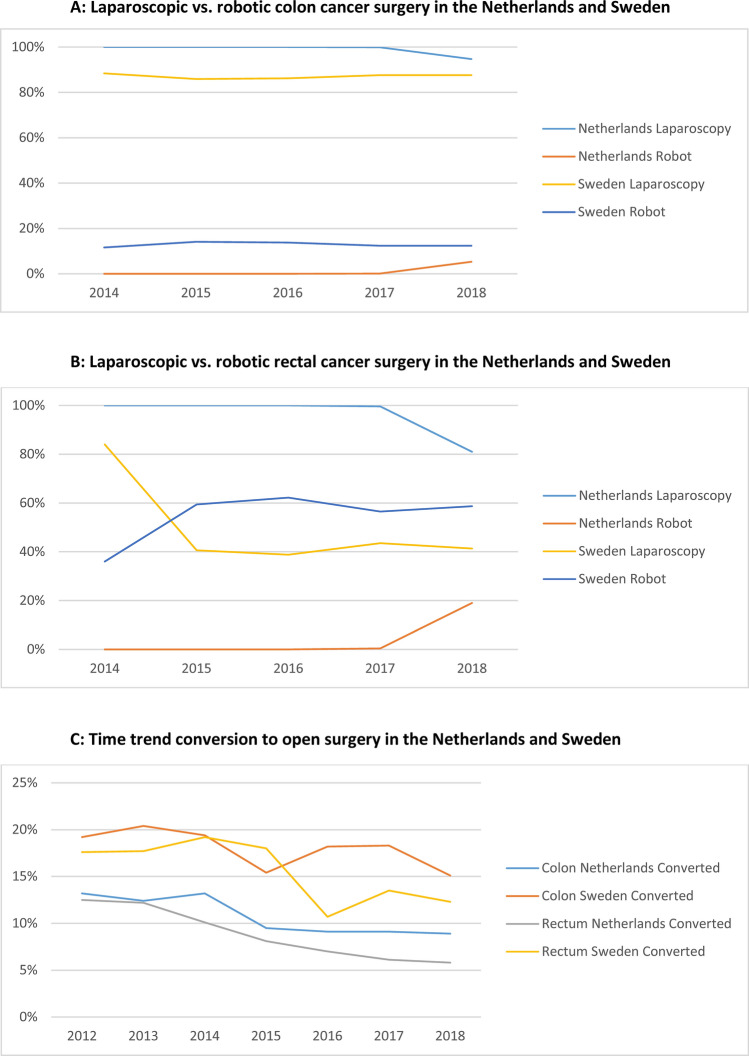
**A** Time trend (2014–2018) laparoscopic vs. robotic surgery for colon cancer in the Netherlands and Sweden. **B** Time trend (2014–2018) laparoscopic vs. robotic surgery for rectal cancer in the Netherlands and Sweden. **C** Time trend (2012–2018) converted laparoscopic vs. robotic surgery for colon and rectal cancer in the Netherlands and Sweden

Regarding the type of MIS used, Fig. 3 demonstrates that robot-assisted laparoscopic surgery for both T1-3 stage colon (Fig. 3A) and rectal cancer (Fig. 3B) was rapidly implemented since 2014 in Sweden (no data available for 2012–2013). Robotic rectal cancer surgery surpassed conventional laparoscopy in 2015 in Sweden, and in 2018, 58.7% of all MIS rectal cancer resections were performed by the robot vs. 41.3% by conventional laparoscopy. Corresponding rates for 2018 in the Netherlands were 19.0% vs. 81.0%, respectively (no data available for 2012–2017 in the DCRA).

In Sweden, conversion rates to open surgery were higher for both colon- and rectal cancer during the entire study period (Fig. 3C). In the Netherlands, the conversion rate to open surgery for colon cancer was decreasing from 13.2% to 9.5% during 2012–2015, and stabilizing thereafter at 8.9% (2018). For rectal cancer, the conversion rate decreased from 12.5% to 5.8% during 2012–2018. In Sweden, conversion rates decreased from 18.8% to 15.5% for colon cancer and from 18.2% to 11.8% for rectal cancer.

### Study population characteristics

Patients that underwent MIS for colon and rectal cancer in Sweden were older, (age ≥ 80: 26.7% vs. 17.0% for colon cancer and 13.9% vs. 11.8% for rectal cancer), more often had an ASA-score III + (30.5% vs. 23.4% for colon cancer and 21.4% vs. 17.0% for rectal cancer), and less frequently a BMI of ≥ 30 (7.5% vs. 20.5% for colon cancer and 6.4% vs. 16.3% for rectal cancer) compared to Dutch patients (Table 1). The clinical stage of colon cancer on CT-imaging was less often defined by Dutch radiologists than by Swedish radiologists (61.3% vs. 22.9% cTx and 48.5% vs. 8.8% cNx), but pathologists defined pT- and pN-stages in comparable proportions. Similar clinical and pathological T- and N-stages were found in rectal cancer patients, but a higher proportion of Dutch rectal cancer patients received neoadjuvant chemoradiation than Swedish patients (27.4% vs. 7.3%). In contrast, Dutch rectal cancer patients received less frequent SCRT than Swedish patients (33.3% vs. 44.1%). Swedish colon cancer patients more often underwent a right hemicolectomy (53.5% vs. 44.4%) and less often a left hemicolectomy (6.0% vs. 11.0%) or transversectomy (0.4% vs. 1.7%). Compared to Dutch rectal cancer patients, a higher proportion of Swedish patients underwent an APE (36.2% vs. 22.2%), and a protective stoma was more frequent in the case of primary anastomosis (64.9% vs. 40.1%).

### Short-term outcomes after MIS

Short-term outcomes after MIS showed a higher proportion of incomplete resection in Sweden than in the Netherlands: 1.7% vs. 0.8% for colon cancer and 6.8% vs. 4.2% for rectal cancer (Table 1). The complication rate, reoperation rate, readmission rate, and mortality rate revealed minimal differences between the two countries.

**Fig. 4:**
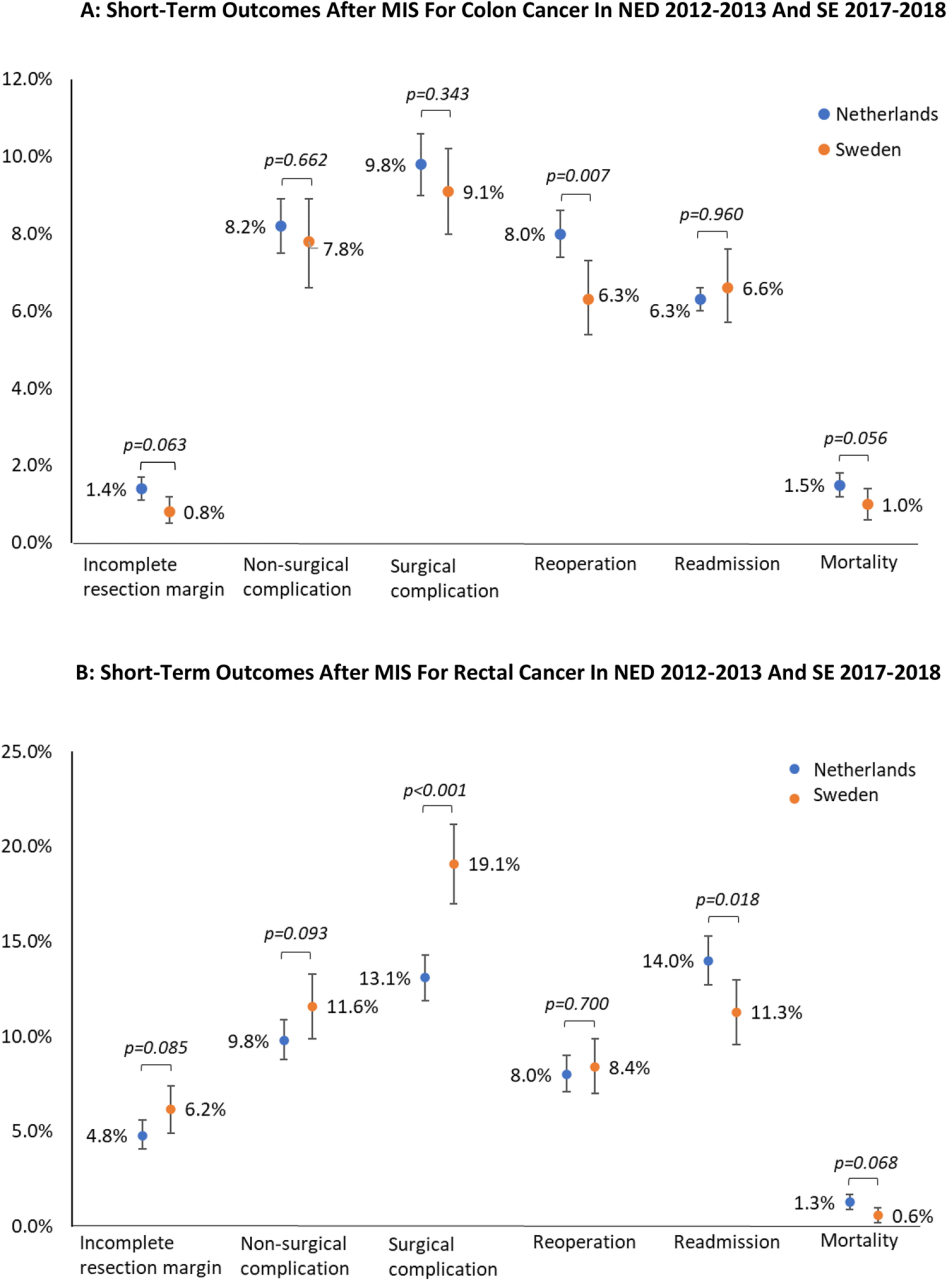
**A** Short term outcome after elective minimal invasive surgery for colon cancer in the Netherlands (2012–2013) (*n* = 6255) and Sweden (2017–2018) (*n* = 2503). **B** Short term outcome after elective minimal invasive surgery for rectal cancer in the Netherlands (2012–2013) (*n* = 2996) and Sweden (2017–2018) (*n* = 1348)

Since Sweden implemented MIS approximately 5 years later than the Netherlands (Fig. 1), different 2-year periods were compared. Therefore, outcomes after MIS in the Netherlands during 2012–2013 were compared with the outcomes after MIS in Sweden during 2017–2018. This resulted in the inclusion of 13,192 Dutch patients (*n* = 6255 colon and *n* = 2996 rectal cancer patients) and 6444 Swedish patients (*n* = 2503 colon cancer and *n* = 1348 rectal cancer patients). The Dutch colon cancer subgroup (2012–2013) showed a higher rate of reoperations (8.0% vs. 6.3%, *p* = 0.007) compared to the Swedish colon cancer subgroup (2017–2018) (Fig. 4A). In the rectal cancer subgroup, a higher rate of surgical complications was found for Sweden (19.1% vs. 13.1%, *p* < 0.001), whereas the readmission rate was higher in the Netherlands (14.0% vs. 11.3%, *p* = 0.018) (Fig. 4B).

### Factors associated with adverse outcomes after MIS

**Fig. 5:**
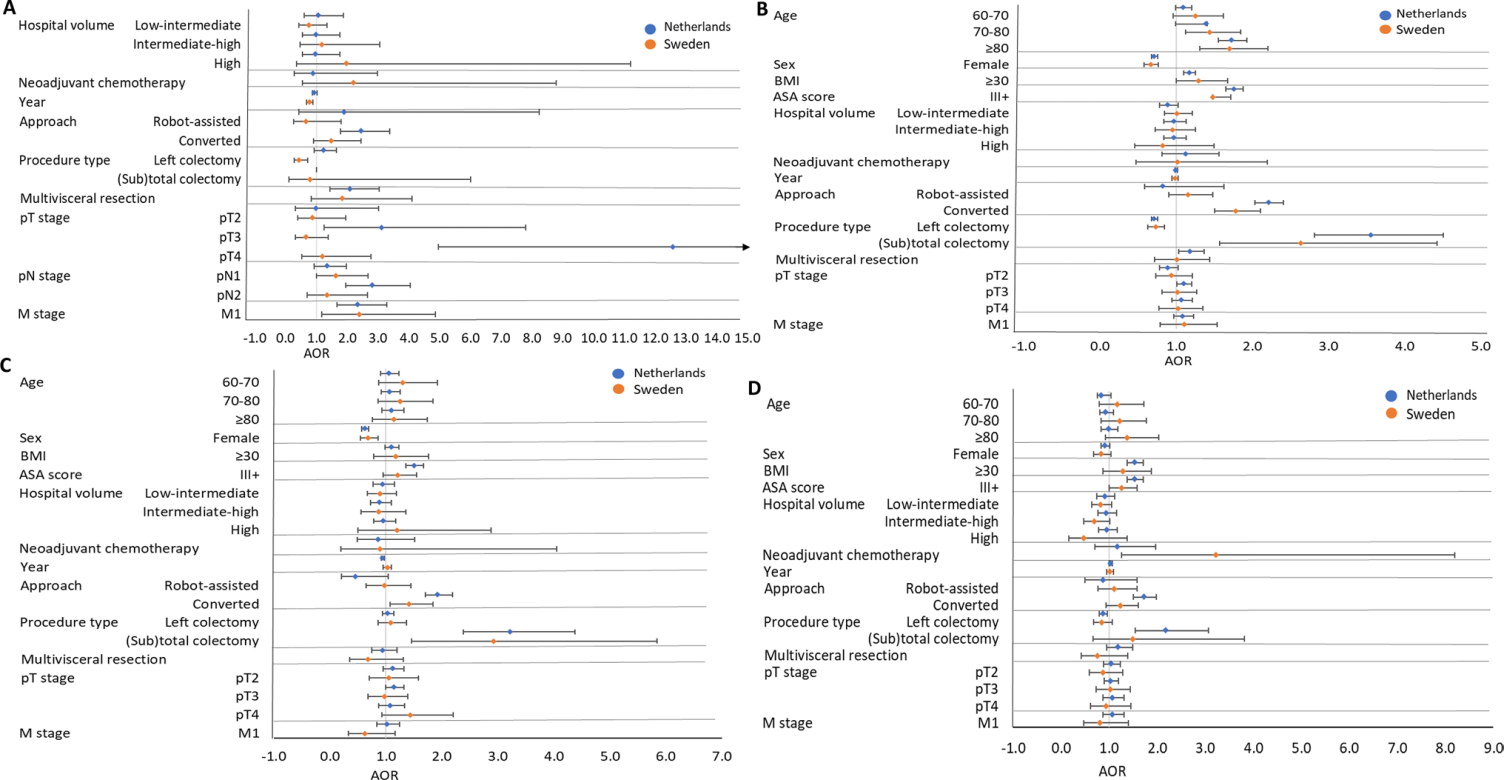
**A** Forest plots of incomplete resection margin after MIS for cT-3 colon cancer. **B** Forest plot of overall complications after MIS for cT-3 colon cancer. **C** Forest plot of reoperations after MIS for cT-3 colon cancer. **D** Forest plot of readmission after MIS for cT-3 colon cancer. *AOR* Adjusted Odds Ratio. Error bars present the 95% CI. The reference category and exact AOR with 95% CI can be found in Supplementary Table S1

**Fig. 6:**
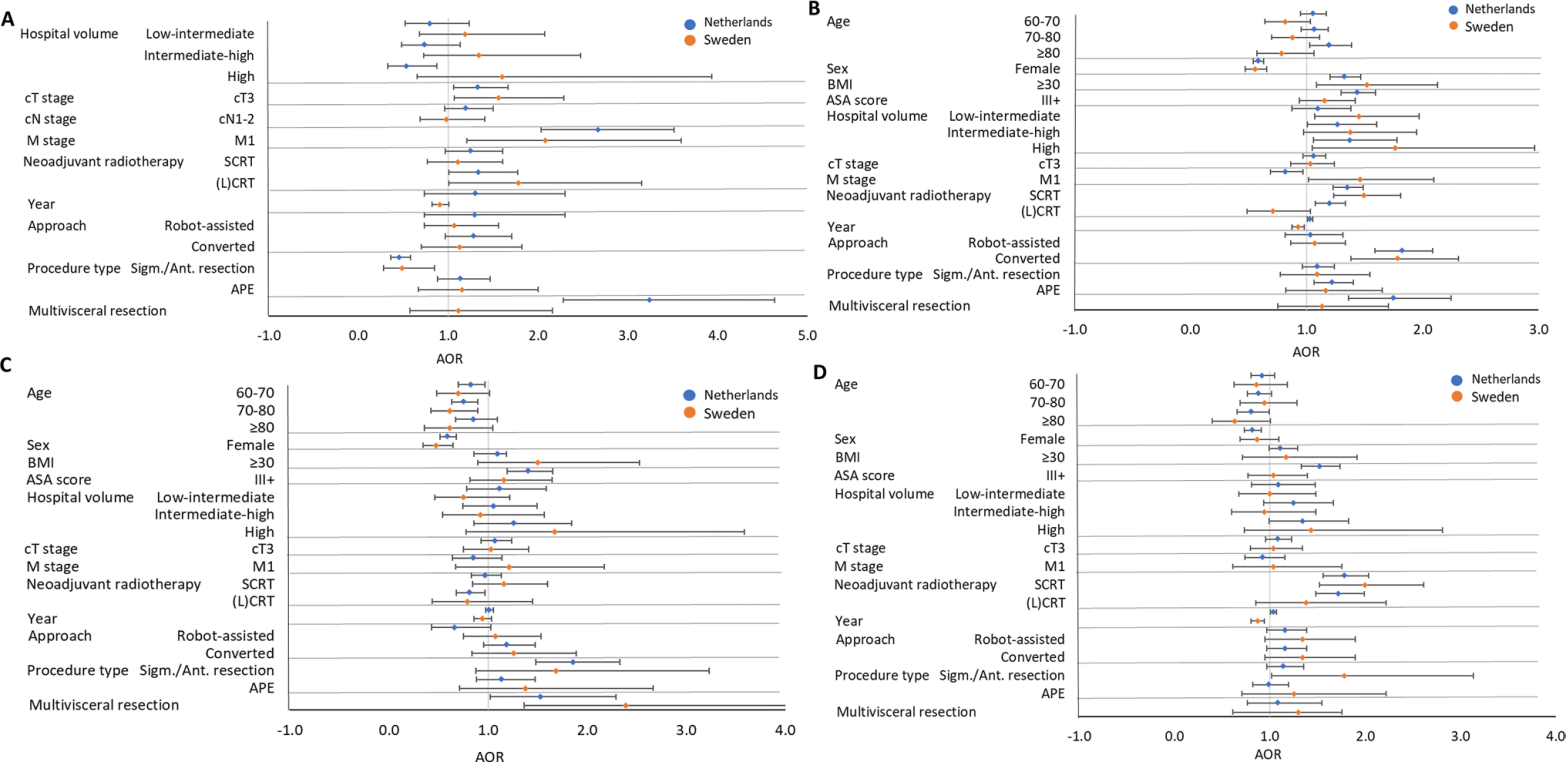
**A** Forest plots of incomplete resection margin after MIS for cT-3 rectal cancer. **B** Forest plot of overall complications after MIS for cT-3 rectal cancer. **C** Forest plot of reoperations after MIS for cT-3 rectal cancer. **D** Forest plot of readmission after MIS for cT-3 rectal cancer. *AOR* Adjuster Odds Ratio. Error bars present the 95% CI. The reference category and exact AOR with 95% CI can be found in Supplementary Table S2

The results of the multilevel logistic regression analyses of factors associated with short-term outcomes after surgery for cT1-3 colon cancer are presented in Fig. 5 and Supplementary Table 1 and for cT1-3 rectal cancer surgery in Fig. 6 and Supplementary Table 2. For the colon cancer population, M-stage was found as a common risk factor for incomplete colon cancer resection for both countries after adjusting for confounders [AOR 2.346, (NED) vs. AOR 2.397 (SE)]. In the Dutch colon cancer patients, conversion to open surgery (AOR 2.465), multivisceral resection (AOR 2.097), pT3- and pT4-stage [AOR 3.131 (pT3) and AOR 12.576 (pT4)], and pN2-stage (AOR 2.821) were identified as risk factors for incomplete resection. In Sweden, the year of surgery (AOR 0.782), pN1-stage (AOR 1.644), and left-sided resections (AOR 0.451) were significantly associated with a decreased risk of incomplete resection. Common risk factors for overall complications after MIS for colon cancer resection in both countries were age ≥ 80 years [AOR 1.732 (NED) and AOR 1.440 (SE)], BMI ≥ 30 [AOR 1.175 (NED) and AOR 1.298 (SE)], ASA-score III + [AOR 1.760 (NED) and AOR 1.480 (SE)], (sub)total colectomy [AOR 3.561 (NED) and AOR 2.639 (SE)] and conversion to open surgery [AOR 2.215 (NED) and AOR 1.783 (SE)]. Female sex [AOR 0.716 (NED) and AOR 0.669 (SE)] and left hemicolectomy [AOR 0.717 (NED) and AOR 0.732 (SE)] decreased the risk. Additional risk factors for overall complication in the Dutch population were multivisceral resections (AOR 1.188) and pT3-stage (AOR 1.101) and in the Sweden population, age 70–80 years (AOR 1.440). Both Dutch and Swedish colon cancer patients showed an increased risk of reoperation after subtotal colectomy [AOR 3.226 (NED) and AOR 2.924 (SE)] and after conversion to open surgery [AOR 1.929 (NED) and AOR 1.408 (SE)]. A decreased reoperation risk was found for female patients [AOR 0.628 (NED) and AOR 0.682 (SE)]. Only Dutch colon cancer patients demonstrated additional factors associated with reoperation, which were ASA-score III + (AOR 1.505) and year of surgery (AOR 0.944). No common risk factors were found for readmission for the colon cancer population.

In the rectal cancer population, common risk factors for incomplete resection among both countries after T1-3 rectal cancer MIS were cT3-stage [AOR 1.327 (NED) and AOR 1.561 (SE)], M1-stage [AOR 2.670 (NED) and AOR 2.080 (SE)], and (chemo)radiotherapy [AOR 1.336 (NED) and AOR 1.782 (SE)]. Additional risk factors in the Netherlands were sigmoid/anterior resection (AOR 0.454) and a multivisceral resection (AOR 3.245) (Suppl. Table 2). Frequent common risk factors for adverse outcomes (i.e., overall complications, reoperations, and readmissions) were after MIS for T1-3 rectal cancer in both countries were BMI ≥ 30, female sex, short-course radiotherapy, and conversion to open surgery. Over time, the risk of overall complications and readmissions for MIS of T1-3 rectal cancer in the Netherlands increased [AOR 1.028 (overall compl.) and AOR 1.037 (readmission)]. In contrast, a decreased risk of overall complications (AOR 0.925) and readmission (AOR 0.871) over the years was observed in Sweden. Additional risk factors for adverse outcomes in the Netherlands were ASA-score III + , (chemo)radiotherapy, procedure type, and multivisceral resection. A hospital volume effect was found for overall complications, with increasing risk in Dutch intermediate-high (AOR 1.269) and high volume hospitals (AOR 1.372) and an increased risk for Swedish low-intermediate (AOR 1.449) and high volume hospitals (AOR 1.763). Besides, Dutch high-volume hospitals showed a decreased risk (AOR 0.535) of incomplete resection.

## Discussion

The present collaborative study evaluated the differences in uptake and outcomes of MIS for colorectal cancer between the Netherlands and Sweden. The implementation of MIS in the Netherlands was approximately 5 years earlier than in Sweden. Over time the number and volume of hospitals performing MIS in Sweden increased, reflecting the first stages of implementing a new surgical modality. Although in the Netherlands an increasing hospital volume for rectal cancer MIS was observed, the number of hospitals decreased. This is most likely the result of rectal cancer care centralization. Despite more low-volume hospitals and an earlier stage of MIS implementation in Sweden, the overall short-term outcomes were comparable with the Netherlands. Even better results for some of the outcomes in Swedish patients were found in the subgroup analysis of the 2 years with a similar stage of implementation. This indicates an appropriate and safe implementation of MIS in Sweden. The substantially higher proportion of robot-assisted surgery in Sweden might have contributed to these findings.

Multiple randomized controlled trials (e.g., COREAN, COLOR, COLOR II, CLASSIC trial, ACOSOG Z6051, ALACART) [9–16] found similar intraoperative complication rates and postoperative morbidity and mortality rates for open and laparoscopic surgery. However, it remains questionable how these RCTs can be translated to implementation at a population level. Nevertheless, several extensive cohort studies [5, 11, 17, 18, 26] have demonstrated favourable results for laparoscopic surgery, such as lower morbidity and mortality rates in high-risk patients [11, 26]. Furthermore, a reduced risk of adhesion-related small bowel obstruction and incisional hernia has been reported [27, 28]. Several technical challenges of laparoscopic surgery have potentially influenced the speed and degree of MIS implementation, including techniques for haemostasis, two-dimensional image, a limited field of view, restricted range of motion, and minimal tactile feedback. These difficulties have led to technological developments, for instance, high-definition three-dimensional optics (e.g., robotic surgery) and advanced energy devices. Nevertheless, conventional laparoscopy has been optimized, and the additional value of robotic-assisted surgery still remains controversial. Several studies failed to show better short-term outcomes and pathological outcomes in robotic-assisted surgery [29, 30], but some small trials showed improved preservation of bladder and sexual function in rectal cancer [31–34]. Robot-assisted surgery also aimed to facilitate the use of MIS in complex colorectal surgery [35], but there are some remaining technical difficulties, especially in multi-quadrant surgery [36, 37].

MIS had a later introduction in Sweden, but the emergence of robot-assisted laparoscopy was much earlier and faster than in the Netherlands. These facts suggest that the timing of MIS implementation was associated with the introduction of robot-assisted surgery in Sweden. The introduction of robotic surgery in Sweden was driven mainly by the demand to recruit urologists and resulted in sudden access to robotic surgery for colorectal surgeons. As a result, many Swedish colorectal surgeons switched directly from open to robotic surgery without taking up conventional laparoscopy.

A well-known advantage of robotic surgery is a shorter learning curve compared to conventional laparoscopic surgery [38–41]. Darcy et al. found that short-term outcomes after robotic surgery already improved after 15 robotic cases [42]. Besides, the improvement in technology led to the da Vinci Xi robot-system, which is a further development of the Si system [36, 37, 43]. In Sweden, the Xi system is now frequently used, and this may have been an advantage when implementing MIS despite lower volumes, especially with the immediate transition from open surgery.

Despite lower volumes in Sweden, a hospital volume effect was not found for most outcomes. This finding might be explained by the standardized training programs and the higher volume of procedures per surgeon as required for robot-assisted laparoscopic surgery, resulting in a relatively short learning curve. In addition, case-mix likely differs between small and high volume hospitals, where complex and high-risk patients are more frequently referred to higher volume (e.g., specialized) centres. The later implementation of MIS in Sweden might have been an advantage, with robotic training programs and access to proctors, and in general, a limited number of colorectal surgeons at each hospital taking part in these programs. However, it should be mentioned that complication rates depend on manual reporting in every individual patient, which might have caused registration bias.

MIS conversion to open surgery rate is often used to assess the MIS learning curve [38] because conversions are mainly associated with hospital volume [44], case complexity, and the experience of the surgeon [38, 45, 46]. We found a higher conversion rate to open surgery in Sweden than in the Netherlands, but with comparable short-term outcomes. These good oncological and surgical results in Sweden for conversion (and multivisceral resections in rectal cancer surgery) might be the influence of the more frequent use of robot-assisted surgical procedures and the more extended experience with open procedures. This suggestion is supported by previous studies which have shown that surgeons early in the MIS learning curve might achieve similar results as experienced surgeons if patient selection (e.g., case complexity) is according to the surgeons’ experience with MIS, thereby stating that conversion rate inadequately reflects the learning curve [38, 46, 47].

In line with a previous collaborative project, we found that Dutch and Swedish patients had different patient-, tumour-, and surgical characteristics [8]. In addition, cT- and cN-stages in colon cancer were more often defined by Swedish radiologists, despite the previously published unreliability of CT imaging for this purpose [48, 49]. However, this had no clinical consequences, given similar proportions of neoadjuvant chemotherapy and pathological disease stages. Mainly due to differences in treatment traditions, neoadjuvant treatment in the form of radio-chemotherapy was given in a lower proportion in Sweden as compared with the Netherlands. Recently the importance of clinical lymph node stage has been tuned down in Swedish guidelines [50], as in contrast to Dutch guidelines [51].

Several limitations of the present study need to be addressed. Robot-assisted surgery is registered in the DCRA since 2018, which has caused an underestimation of the number of robotic procedures. In Sweden, one hospital started in 2010 with robotic surgery and, several other hospitals started in 2013–2014, whereas the first Dutch hospital started in 2011. Several Swedish hospitals performed only one MIS procedure for rectal cancer per year. This finding may be due to an incorrect diagnosis (distal sigmoid colon instead of the upper rectum) or possibly by a procedure performed by a guest surgeon. Data might be differently registered in the SCRCR and DCRA due to national differences in usage and perception of the registries. For example, definitions of postoperative complications might differ, and complications might be more thoroughly registered in one of the registry’s recordings resulting in reduced comparability. In addition, details on individual complications are lacking in the DCRA. Besides, the Dutch and Swedish populations differed from each other, most likely due to patient selection. The selection bias might result in different outcomes. However, selection of the right patient for a surgical procedure also depends on the appropriate application of MIS. As a result of the retrospective character of the study, the mortality rate might not be an appropriate outcome parameter due to the significant historical improvement over time as a consequence of optimized perioperative care.

The present collaborative research project showed a delay in implementation of MIS of 5 years in Sweden when compared to the Netherlands, but with good results despite relatively low volumes. The absence of a hospital volume effect in Sweden suggests a beneficial role of late adaption with the maturation of a new technique, besides a potential impact of the immediate switch from open to robot-assisted laparoscopy. The good oncological and surgical results in Sweden after conversion in combination with higher conversion rates if compared to the Netherlands illustrates the safe implementation of MIS in Sweden. The results indicate that later implementation of MIS might have advantages and that access to robotic training programs and proctors might overcome learning curve issues in case of lower volumes.

## Supplementary Information

Below is the link to the electronic supplementary material.

Supplementary file1 (DOCX 36 KB)
