# Supplementary material for: National differences in implementation of minimally invasive surgery for colorectal cancer and the influence on short-term outcomes
Source: Surg Endosc. 2022 Mar 8;36(8):5986–6001. doi: 10.1007/s00464-021-08974-1 (PMC9283170; doi:10.1007/s00464-021-08974-1)
Supplement: Supplementary file 1 — Supplementary file1 (DOCX 36 KB) [file 464_2021_8974_MOESM1_ESM.docx]

**SUPPLEMENTARY TABLE 1: Multilevel Logistic Analyses Of MIS For T1-3 Colon Cancer In The Netherlands And Sweden**

|  |  | **Incomplete resection** | | | **Overall complications** | | **Reoperation** | | **Readmission** | |
| --- | --- | --- | --- | --- | --- | --- | --- | --- | --- | --- |
|  |  | **NED (N=29,655)** | | **SE (N=5,660)** | **NED (N=29,849)** | **SE (N=5,685)** | **NED (N=29,804)** | **SE (N=5,682)** | **NED (N=29,359)** | **SE (N=5,676)** |
|  |  | AOR (95% CI) | | AOR (95% CI) | AOR (95% CI) | AOR (95% CI) | AOR (95% CI) | AOR (95% CI) | AOR (95% CI) | AOR (95% CI) |
| **Age** | <60 |  |  | | 1.00 (Ref.) | 1.00 (Ref.) | 1.00 (Ref.) | 1.00 (Ref.) | 1.00 (Ref.) | 1.00 (Ref.) |
|  | 60-70 |  |  | | 1.094 (0.992-1.207) | 1.251 (0.966-1.620) | 1.056 (0.904-1.232) | 1.294 (0.871-1.923) | 0.883 (0.756-1.032) | 1.161 (0.785-1.718) |
|  | 70-80 |  |  | | 1.393  (0.992-1.407) | 1.440 (1.124-1.844) | 1.073 (0.920-1.250) | 1.256 (0.855-1.845) | 0.933 (0.801-1.087) | 1.215 (0.835-1.768) |
|  | ≥80 |  |  | | 1.732 (1.556-1.929) | 1.700 (1.310-2.205) | 1.106 (0.927-1.320) | 1.144 (0.753-1.737) | 0.989 (0.830-1.180) | 1.368 (0.921-2.033) |
| **Sex** | Male |  |  | | 1.00 (Ref.) | 1.00 (Ref.) | 1.00 (Ref.) | 1.00 (Ref.) | 1.00 (Ref.) | 1.00 (Ref.) |
|  | Female |  |  | | 0.716 (0.675-0.758) | 0.669 (0.582-0.770) | 0.628 (0.570-0.693) | 0.682 (0.543-0.858) | 0.920 (0.835-1.014) | 0.832 (0.671-1.032) |
| **BMI** | <30 |  |  | | 1.00 (Ref.) | 1.00 (Ref.) | 1.00 (Ref.) | 1.00 (Ref.) | 1.00 (Ref.) | 1.00 (Ref.) |
|  | ≥30 |  |  | | 1.175 (1.097-1.258) | 1.298 (1.002-1.681) | 1.096 (0.979-1.227) | 1.175 (0.783-1.764) | 1.029 (0.915-1.157) | 1.276 (0.866-1.881) |
| **ASA** | I-II |  |  | | 1.00 (Ref.) | 1.00 (Ref.) | 1.00 (Ref.) | 1.00 (Ref.) | 1.00 (Ref.) | 1.00 (Ref.) |
|  | III+ |  |  | | 1.760 (1.650-1.878) | 1.480 (1.480-1.714) | 1.505 (1.353-1.674) | 1.211 (0.951-1.542) | 1.528 (1.370-1.705) | 1.251 (0.995-1.573) |
| **Hospital volume*** | Low | 1.00 (Ref.) | 1.00 (Ref.) | | 1.00 (Ref.) | 1.00 (Ref.) | 1.00 (Ref.) | 1.00 (Ref.) | 1.00 (Ref.) | 1.00 (Ref.) |
|  | Low-intermediate | 1.064 (0.600-1.886) | 0.761 (0.429-1.348) | | 0.897 (0.781-1.031) | 1.014 (0.849-1.212) | 0.941 (0.772-1.148) | 0.894 (0.673-1.188) | 0.908 (0.738-1.117) | 0.819 (0.641-1.046) |
|  | Intermediate-high | 0.987 (0.553-1.763) | 1.194 (0.467-3.053) | | 0.975 (0.840-1.133) | 0.957 (0.730-1.253) | 0.891 (0.726-1.092) | 0.869 (0.559-1.351) | 0.938 (0.763-1.154) | 0.687 (0.469-1.005) |
|  | High | 0.984 (0.548-1.768) | 1.980 (0.350-11.212) | | 0.975 (0.840-1.133) | 0.826 (0.456-1.497) | 0.958 (0.778-1.179) | 1.200 (0.501-2.874) | 0.949 (0.773-1.166) | 0.474 (0.164-1.372) |

| **Neoadj. chemo** | No | 1.00 (Ref.) | 1.00 (Ref.) | 1.00 (Ref.) | 1.00 (Ref.) | 1.00 (Ref.) | 1.00 (Ref.) | 1.00 (Ref.) | 1.00 (Ref.) |
| --- | --- | --- | --- | --- | --- | --- | --- | --- | --- |
|  | Yes | 0.906 (0.275-2.984) | 2.207 (0.555-8.779) | 1.128 (0.813-1.566) | 1.019 (0.473-2.198) | 0.862 (0.489-1.518) | 0.895 (0.198-4.045) | 1.172 (0.697-1.971) | 3.214 (1.260-8.197) |
| **Year** |  | 0.943 (0.871-1.021) | 0.782 (0.684-0.893) | 0.999 (0.983-1.016) | 0.985 (0.942-1.029) | 0.944 (0.919-0.970) | 1.023 (0.952-1.100) | 1.033 (1.004-1.062) | 1.016 (0.952-1.085) |
| **Approach** | Laparoscopic | 1.00 (Ref.) | 1.00 (Ref.) | 1.00 (Ref.) | 1.00 (Ref.) | 1.00 (Ref.) | 1.00 (Ref.) | 1.00 (Ref.) | 1.00 (Ref.) |
|  | Robot | 1.907 (0.442-8.228) | 0.680 (0.256-1.812) | 0.826 (0.586-1.627) | 1.156 (0.902-1.482) | 0.457 (0.201-1.040) | 0.969 (0.649-1.447) | 0.878 (0.500-1.575) | 1.102 (0.769-1.579) |
|  | Converted | 2.465 (1.793-3.390) | 1.492 (0.908-2.451) | 2.215 (2.037-2.408) | 1.783 (1.509-2.106) | 1.929 (1.701-2.188) | 1.408 (1.075-1.845) | 1.725 (1.508-1.974) | 1.225 (0.937-1.600) |
| **Procedure type** | Right | 1.00 (Ref.) | 1.00 (Ref.) | 1.00 (Ref.) | 1.00 (Ref.) | 1.00 (Ref.) | 1.00 (Ref.) | 1.00 (Ref.) | 1.00 (Ref.) |
|  | Left | 1.241 (0.936-1.643) | 0.451 (0.284-0.718) | 0.717 (0.677-0.760) | 0.732 (0.632-0.847) | 1.037 (0.942-1.142) | 1.085 (0.860-1.369) | 0.872 (0.790-0.963) | 0.844 (0.674-1.057) |
|  | (Sub)total | - | 0.796 (0.105-6.007) | 3.561 (2.813-4.506) | 2.639 (1.575-4.420) | 3.226 (2.376-4.379) | 2.924 (1.462-5.846) | 2.179 (1.546-3.070) | 1.494 (0.661-3.82) |
| **Multivisceral resection** | No | 1.00 (Ref.) | 1.00 (Ref.) | 1.00 (Ref.) | 1.00 (Ref.) | 1.00 (Ref.) | 1.00 (Ref.) | 1.00 (Ref.) | 1.00 (Ref.) |
|  | Yes | 2.097 (1.450-3.031) | 1.846 (0.829-4.110) | 1.188 (1.034-1.366) | 1.015 (0.717-1.438) | 0.946 (0.749-1.195) | 0.680 (0.353-1.310) | 1.186 (0.945-1.488) | 0.758 (0.414-1.385) |
| **pT stage** | T1 | 1.00 (Ref.) | 1.00 (Ref.) | 1.00 (Ref.) | 1.00 (Ref.) | 1.00 (Ref.) | 1.00 (Ref.) | 1.00 (Ref.) | 1.00 (Ref.) |
|  | T2 | 0.987 (0.322-3.022) | 0.879 (0.397-1.948) | 0.897 (0.781-1.031) | 0.946 (0.735-1.218) | 1.121 (0.952-1.320) | 1.056 (0.707-1.579) | 1.045 (0.884-1.234) | 0.867 (0.586-1.282) |
|  | T3 | 3.131 (1.257-7.795) | 0.669 (0.325-1.379) | 1.101 (1.008-1.202) | 1.016 (0.814-1.269) | 1.145 (0.990-1.324) | 0.974 (0.680-1.394) | 1.031 (0.889-1.196) | 1.023 (0.729-1.436) |
|  | T4 | 12.576 (4.954-31.924) | 1.206 (0.524-2.776) | 1.074 (0.948-1.217) | 1.027 (0.777-1.356) | 1.079 (0.876-1.329) | 1.431 (0.932-2.198) | 1.066 (0.864-1.314) | 0.938 (0.607-1.449) |
| **pN stage** | N0 | 1.00 (Ref.) | 1.00 (Ref.) |  |  |  |  |  |  |
|  | N1 | 1.354 (0.932-1.967) | 1.644 (1.010-2.675) |  |  |  |  |  |  |
|  | N2 | 2.821 (1.965-4.050) | 1.359  (0.692-2.669) |  |  |  |  |  |  |
| **M stage** | M- | 1.00 (Ref.) | 1.00 (Ref.) | 1.00 (Ref.) | 1.00 (Ref.) | 1.00 (Ref.) | 1.00 (Ref.) | 1.00 (Ref.) | 1.00 (Ref.) |
|  | M1 | 2.346 (1.671-3.293) | 2.397 (1.181-4.867) | 1.092 (0.967-1.233) | 1.106 (0.795-1.538) | 1.020 (0.836-1.244) | 0.617 (0.328-1.158) | 1.068 (0.871-1.310) | 0.805 (0.463-1.402) |

Supplementary table 1 presents the results of the multilevel logistic regression analyses reported by the adjusted odds ratio (AOR) and 95% confidence interval (95% CI). Variables that were associated with a pathologic- or surgical outcomes after MIS of T1-3 colon cancer are highlighted bold. NED: Netherlands, SE: Sweden, neoadj. chemo: neoadjuvant chemotherapy, procedure right sided: ileocecal resections, (extended) right hemicolectomies and transversectomies, procedure left sided: (extended) left hemicolectomies, sigmoid/anterior resections, Hartmann procedures and the (sub)total colectomy group, procedure type (sub)total: (sub)total colectomy. *Hospital volume: low volume <30, low-intermediate volume 30-60, intermediate-high volume 61-90, high volume >90 colon cancer resections.

**SUPPLEMENTARY TABLE 2: Multilevel Logistic Analyses Of MIS For T1-3 Rectal Cancer In The Netherlands And Sweden**

|  |  | **Incomplete resection** | | **Overall complications** | | **Reoperation** | | **Readmission** | |
| --- | --- | --- | --- | --- | --- | --- | --- | --- | --- |
|  |  | **NED (N=12,107)** | **SE (N=2,695)** | **NED (N=12,996)** | **SE (N=2,780)** | **NED (N=12,968)** | **SE (N=2,781)** | **NED (N=12,779)** | **SE (N=2,776)** |
|  |  | AOR (95% CI) | AOR (95% CI) | AOR (95% CI) | AOR (95% CI) | AOR (95% CI) | AOR (95% CI) | AOR (95% CI) | AOR (95% CI) |
| **Age** | <60 |  |  | 1.00 (Ref.) | 1.00 (Ref.) | 1.00 (Ref.) | 1.00 (Ref.) | 1.00 (Ref.) | 1.00 (Ref.) |
|  | 60-70 |  |  | 1.054 (0.949-1.170) | 0.814 (0.642-1.032) | 0.825 (0.701-0.970) | 0.700 (0.481-1.018) | 0.917 (0.799-1.052) | 0.859 (0.624-1.182) |
|  | 70-80 |  |  | 1.064 (0.954-1.185) | 0.880 (0.697-1.110) | 0.757 (0.637-0.899) | 0.616 (0.423-0.897 | 0.881 (0.763-1.017) | 0.940 (0.688-1.284) |
|  | ≥80 |  |  | 1.194 (1.028-1.387) | 0.781 (0.573-1.065) | 0.857 (0.671-1.091) | 0.615 (0.360-1.051) | 0.806 (0.655-0.991) | 0.629 (0.392-1.008) |
| **Sex** | Male |  |  | 1.00 (Ref.) | 1.00 (Ref.) | 1.00 (Ref.) | 1.00 (Ref.) | 1.00 (Ref.) | 1.00 (Ref.) |
|  | Female |  |  | 0.583 (0.537-0.632) | 0.554 (0.468-0.657) | 0.591 (0.514-0.678) | 0.474 (0.348-0.645) | 0.818 (0.733-0.913) | 0.867 (0.685-1.097) |
| **BMI** | <30 |  |  | 1.00 (Ref.) | 1.00 (Ref.) | 1.00 (Ref.) | 1.00 (Ref.) | 1.00 (Ref.) | 1.00 (Ref.) |
|  | ≥30 |  |  | 1.328 (1.204-1.466) | 1.520 (1.084-2.131) | 1.100 (0.855-1.186) | 1.504 (0.894-2.530) | 1.130 (0.990-1.289) | 1.170 (0.714-1.917) |
| **ASA** | I-II |  |  | 1.00 (Ref.) | 1.00 (Ref.) | 1.00 (Ref.) | 1.00 (Ref.) | 1.00 (Ref.) | 1.00 (Ref.) |
|  | III+ |  |  | 1.437 (1.298-1.592) | 1.154 (0.939-1.419) | 1.406 (1.195-1.654) | 1.160 (0.818-1.646) | 1.521 (1.331-1.738) | 1.036 (0.770-1.394) |
| **Hospital volume** | Low | 1.00 (Ref.) | 1.00 (Ref.) | 1.00 (Ref.) | 1.00 (Ref.) | 1.00 (Ref.) | 1.00 (Ref.) | 1.00 (Ref.) | 1.00 (Ref.) |
|  | Low-intermediate | 0.797 (0.516-1.231) | 1.189 (0.681-2.074) | 1.099 (0.874-1.383) | 1.449 (1.067-1.968) | 1.114 (0.782-1.588) | 0.755 (0.466-1.222) | 1.094 (0.811-1.476) | 0.997 (0.672-1.479) |
| Intermediate -high | | 0.738 (0.481-1.133) | 1.341 (0.728-2.469) | 1.269 (1.005-1.602) | 1.378 (0.973-1.951) | 1.055 (0.743-1.498) | 0.921 (0.541-1.569) | 1.248 (0.935-1.666) | 0.944 (0.601-1.484) |
|  | High | 0.535 (0.328-0.873) | 1.599 (0.651-3.932) | 1.372 (1.057-1.780) | 1.763 (1.048-2.965) | 1.259 (0.860-1.843) | 1.671 (0.779-3.586) | 1.347 (0.993-1.827) | 1.432 (0.730-2.809) |
| **cT stage** | T1-2 | 1.00 (Ref.) | 1.00 (Ref.) | 1.00 (Ref.) | 1.00 (Ref.) | 1.00 (Ref.) | 1.00 (Ref.) | 1.00 (Ref.) | 1.00 (Ref.) |
|  | T3 | 1.327 (1.056-1.666) | 1.561 (1.066-2.285) | 1.063 (0.971-1.163) | 1.033 (0.861-1.239) | 1.070 (0.927-1.236) | 1.031 (0.755-1.407) | 1.084 (0.956-1.228) | 1.036 (0.798-1.344) |
| **cN stage** | N0 | 1.00 (Ref.) | 1.00 (Ref.) |  |  |  |  |  |  |
|  | N1-2 | 1.199 (0.960-1.496) | 0.982 (0.686-1.405) |  |  |  |  |  |  |
| **M stage** | M- | 1.00 (Ref.) | 1.00 (Ref.) | 1.00 (Ref.) | 1.00 (Ref.) | 1.00 (Ref.) | 1.00 (Ref.) | 1.00 (Ref.) | 1.00 (Ref.) |
|  | M1 | 2.670 (2.031-3.510) | 2.080 (1.205-3.591) | 0.818 (0.688-0.971) | 1.461 (1.017-2.099) | 0.855 (0.641-1.141) | 1.211 (0.676-2.169) | 0.923 (0.736-1.158) | 1.034 (0.609-1.757) |
| **Neoadj. RTx** | No | 1.00 (Ref.) | 1.00 (Ref.) | 1.00 (Ref.) | 1.00 (Ref.) | 1.00 (Ref.) | 1.00 (Ref.) | 1.00 (Ref.) | 1.00 (Ref.) |
|  | SCRT | 1.247 (0.966-1.608) | 1.109 (0.764-1.609) | 1.353 (1.228-1.490) | 1.495 (1.234-1.811) | 0.972 (0.834-1.133) | 1.162 (0.844-1.600) | 1.781 (1.559-2.036) | 1.994 (1.523-2.611) |
|  | (L)CRT | 1.336 (1.007-1.773) | 1.782 (1.007-3.153) | 1.197 (1.073-1.334) | 0.707 (0.484-1.032) | 0.814 (0.682-0.972) | 0.794 (0.435-1.452) | 1.718 (1.482-1.992) | 1.378 (0.854-2.222) |
| **Year** |  | 1.300 (0.735-2.298) | 0.908 (0.821-1.004) | 1.028 (1.004-1.052) | 0.925 (0.873-0.980) | 1.014 (0.978-1.052) | 0.942 (0.856-1.036) | 1.037 (1.006-1.069) | 0.871 (0.804-0.943) |
| **Approach** | Laparoscopic | 1.00 (Ref.) | 1.00 (Ref.) | 1.00 (Ref.) | 1.00 (Ref.) | 1.00 (Ref.) | 1.00 (Ref.) | 1.00 (Ref.) | 1.00 (Ref.) |
|  | Robot | 1.299 (0.735-2.298) | 1.067 (0.731-1.558) | 1.034 (0.815-1.313) | 1.071 (0.861-1333) | 0.666 (0.430-1.031) | 1.074 (0.752-1.534) | 0.999 (0.730-1.367) | 1.290 (0.955-1.742) |
|  | Converted | 1.284 (0.967-1.705) | 1.128 (0.701-1.816) | 1.822 (1.590-2.086) | 1.788 (1.383-2.311) | 1.188 (0.957-1.475) | 1.260 (0.840-1.891) | 1.159 (0.967-1.389) | 1.340 (0.948-1.895) |
| **Procedure type** | Hartmann | 1.00 (Ref.) | 1.00 (Ref.) | 1.00 (Ref.) | 1.00 (Ref.) | 1.00 (Ref.) | 1.00 (Ref.) | 1.00 (Ref.) | 1.00 (Ref.) |
|  | Sig./AR | 0.454 (0.357-0.579) | 0.487 (0.280-0.846) | 1.094 (0.965-1.240) | 1.094 (0.774-1.546) | 1.859 (1.481-2.332) | 1.686 (0.879-3.232) | 1.142 (0.964-1.353) | 1.782 (1.015-3.131) |
|  | APE | 1.136 (0.879-1.469) | 1.150 (0.663-1.996) | 1.222 (1.065-1.402) | 1.164 (0.821-1.650) | 1.140 (0.882-1.475) | 1.378 (0.712-2.667) | 0.992 (0.822-1.197) | 1.255 (0.708-2.222) |
| **Multivisceral resection** | No | 1.00 (Ref.) | 1.00 (Ref.) | 1.00 (Ref.) | 1.00 (Ref.) | 1.00 (Ref.) | 1.00 (Ref.) | 1.00 (Ref.) | 1.00 (Ref.) |
|  | Yes | 3.245 (2.276-4.629) | 1.114 (0.575-2.157) | 1.749 (1.362-2.246) | 1.131 (0.750-1.704) | 1.531 (1.024-2.289) | 2.386 (1.361-4.180) | 1.087 (0.765-1.543) | 1.299 (0.609-1.757) |

Supplementary table 2 presents the results of the multilevel logistic regression analyses reported by the adjusted odds ratio (AOR) and 95% confidence interval (95% CI). Variables that were associated with a pathologic- or surgical outcomes after MIS of T1-3 rectal cancer are highlighted bold. NED: Netherlands, SE: Sweden, neoadj RTx: neoadjuvant radiotherapy, SCRT: short course radiotherapy, (L)CRT (long-course)chemoradiotherapy, procedure type sig./AR: sigmoid resection/(anterior resection, Hartmann procedure, APE: Abdominoperineal Excision. *Hospital volume: low volume <12, low-intermediate volume 12-25, intermediate-high volume 26-50, high volume >50 rectal cancer resections.
